# Supplementary material for: Dietary risk factors for hip fracture in adults: An umbrella review of meta-analyses of prospective cohort studies
Source: PLoS One. 2021 Nov 10;16(11):e0259144. doi: 10.1371/journal.pone.0259144 (PMC8580223; doi:10.1371/journal.pone.0259144)
Supplement: S1 Table — (DOCX) [file pone.0259144.s001.docx]

**S1 Table: Search strategy.**

| **Ovid Embase (311 articles returned)** | |
| --- | --- |
| 1.Exposure terms | (Nutrient* OR micronutrient* OR macronutrient* OR phytonutrient* OR antioxidant*).tw. OR (exp nutrient/ OR trace element/ OR macronutrient intake/ OR macronutrient/ OR antioxidant/ OR phytonutrient/) OR (vitamin* OR vitamin D OR vitamin K OR vitamin C OR vitamin B* OR thiamine OR riboflavin OR pyridoxine OR cyanocobalamin OR dietary minerals OR iron intake OR zinc intake OR magnesium intake OR sodium intake OR potassium intake OR silicon intake OR boron intake OR calcium intake OR copper intake OR manganese intake OR iodine intake).tw. OR (vitamin B group/ OR vitamin D deficiency/ OR vitamin B deficiency/ OR vitamin deficiency/ OR vitamin K deficiency/ OR vitamin/ OR vitamin intake/ OR vitamin K group/ OR vitamin D/ OR thiamine/ OR riboflavin/ OR pyridoxine/ OR cyanocobalamin/ OR mineral intake/ OR iron intake/ OR zinc intake/ OR magnesium intake/ OR sodium intake/ OR potassium intake/ OR calcium intake/ OR copper intake/ OR iodine intake/) OR (protein intake OR protein consumption OR animal protein OR plant protein OR dietary protein OR dietary fat* OR saturated fat OR mono-unsaturated fatty acids OR poly-unsaturated fatty acids OR MUFAs OR PUFAs OR carbohydrate* OR sugar* OR dietary fibre OR dietary fiber OR energy intake OR calorie intake OR caloric intake).tw. OR (protein intake/ OR animal protein/ OR plant protein/ OR fat intake/ OR monounsaturated fatty acid/ OR unsaturated fatty acid/ OR saturated fatty acid/ OR polyunsaturated fatty acid/ OR carbohydrate intake/ OR sugar intake/ OR dietary fiber/ OR caloric intake/) OR (caffeinated intake OR caffeine intake OR caffeine OR decaffeinated OR decaffeinated intake OR dietary supplement* OR alcohol OR dairy OR milk OR cheese OR yogurt OR coffee OR meat OR fruit* OR vegetable* OR veg OR legumes OR nuts).tw. OR (caffeine intake/ OR diet supplementation/ OR dietary supplement/ OR alcohol consumption/ OR exp dairy product/ OR coffee consumption/ OR meat consumption/ OR processed meat/ OR red meat/ OR fruit consumption/ OR vegetable consumption/ OR food intake/) OR (dietary pattern OR diet OR omnivore OR omnivorous OR vegetarian OR vegan OR plant-based OR plant based OR lacto-vegetarian OR lactovegetarian OR Mediterranean diet OR MedDiet OR Western diet OR dietary approaches to stop hypertension OR DASH diet OR fasting OR dietary risk factor*).tw. OR (dietary pattern/ OR omnivore/ OR exp vegetarian/ OR exp vegetarian diet/ OR vegan diet/ OR vegan/ OR Mediterranean diet/ OR DASH diet/ OR intermittent fasting/ OR fasting/ OR Ramadan fasting/ OR dietary intake/) |
| 2.Primary outcome terms | (hip fracture* OR osteoporotic fracture OR bone fracture OR fragility fracture).tw. OR (hip fracture/ OR fragility fracture/) NOT treatment.tw. |
| 3.Review terms | *BMJ Embase pre-tested search filter for systematic reviews:*   1. exp review/ 2. (literature adj3 review$).ti,ab. 3. exp meta analysis/ 4. exp “Systematic Review”/ 5. or/1-4 6. (medline or medlars or embase or pubmed or cinahl or amed or psychlit or psyclit or psychinfo or psycinfo or scisearch or cochrane).ti,ab. 7. RETRACTED ARTICLE/ 8. 6 or 7 9. 5 and 8 10. (systematic$ adj2 (review$ or overview)).ti,ab. 11. (meta?anal$ or meta anal$ or meta-anal$ or metaanal$ or metanal$).ti,ab. 12. 9 or 10 or 11 |
| 4. Boolean operators | 1 AND 2 AND 3 |
| 5. Limits | Limit 4 to humans  Limit 5 to English Language |
| **Ovid Medline (136 articles returned)** | |
| 1. Exposure terms | (Nutrient* OR micronutrient* OR macronutrient* OR phytonutrient* OR antioxidant*).tw. OR (nutrients/ OR micronutrients/ OR phytochemicals/ OR antioxidants/) OR (vitamin* OR vitamin D OR vitamin K OR vitamin C OR vitamin B* OR thiamine OR riboflavin OR pyridoxine OR cyanocobalamin OR dietary minerals OR iron intake OR zinc intake OR magnesium intake OR sodium intake OR potassium intake OR silicon intake OR boron intake OR calcium intake OR copper intake OR manganese intake OR iodine intake).tw. OR (vitamins/ OR vitamin D/ OR vitamin A deficiency/ OR vitamin B deficiency/ OR vitamin D deficiency/ OR vitamin E deficiency/ OR vitamin K deficiency/ OR thiamine/ OR riboflavin/ OR pyridoxine/ OR vitamin B12/ OR trace elements/ OR iron, dietary/ OR magnesium deficiency/ OR potassium deficiency/ OR sodium, dietary/ OR sodium chloride, dietary/ OR calcium, dietary/) OR (protein intake OR protein consumption OR animal protein OR plant protein OR dietary protein OR dietary fat* OR saturated fat OR mono-unsaturated fatty acids OR poly-unsaturated fatty acids OR MUFAs OR PUFAs OR carbohydrate* OR sugar* OR dietary fibre OR dietary fiber OR energy intake OR calorie intake OR caloric intake).tw. OR (Dietary proteins/ OR animal proteins, dietary/ OR fruit proteins/ OR grain proteins/ OR plant proteins, dietary/ OR dietary fats/ OR butter/ OR cholesterol, dietary/ OR dietary fats, unsaturated/ OR margarine/ OR fats, unsaturated/ OR dietary carbohydrates/ OR dietary fiber/ OR dietary sugars/ OR starch/ OR energy intake/ OR caloric restriction/ OR portion size/ OR serving size/) OR (caffeinated intake OR caffeine intake OR caffeine OR decaffeinated OR decaffeinated intake OR dietary supplement* OR alcohol OR dairy OR milk OR cheese OR yogurt OR coffee OR meat OR fruit* OR vegetable* OR veg OR legumes OR nuts).tw. OR (Caffeine/ OR dietary supplements/ OR alcohol abstinence/ OR alcohol drinking/ OR dairy products/ OR butter/ OR cultured milk products/ OR ice cream/ OR margarine/ OR milk/ OR coffee/ OR meat/ OR meat products/ OR poultry/ OR red meat/ OR seafood/ OR fruit/ OR nuts/ OR seeds/ OR vegetables/) OR (dietary pattern OR diet OR omnivore OR omnivorous OR vegetarian OR vegan OR plant-based OR plant based OR lacto-vegetarian OR lactovegetarian OR Mediterranean diet OR MedDiet OR Western diet OR dietary approaches to stop hypertension OR DASH diet OR fasting OR dietary risk factor*).tw. OR (diet/ OR diet, carbohydrate-restricted/ OR diet, fat-restricted/ OR diet, gluten-free/ OR diet, high-fat/ OR diet, high-protein/ OR diet, Mediterranean/ OR diet, vegetarian/ OR diet, western/ OR dietary approaches to stop hypertension/ OR fasting/ OR vegetarians/ OR vegans/) |
| 1. Primary outcome terms | (Hip fracture* OR osteoporotic fracture OR bone fracture OR fragility fracture).tw. OR (hip fractures/ OR femoral neck fractures/) NOT treatment.tw. |
| 1. Review terms | *BMJ Medline pre-tested search filter for systematic reviews:*   1. review.pt. 2. (medline or medlars or embase or pubmed or cochrane).tw,sh. 3. (scisearch or psychinfo or psycinfo).tw,sh. 4. (psychlit or psyclit).tw,sh. 5. cinahl.tw,sh. 6. ((hand adj2 search$) or (manual$ adj2 search$)).tw,sh. 7. (electronic database$ or bibliographic database$ or computeri?ed database$ or online database$).tw,sh. 8. (pooling or pooled or mantel haenszel).tw,sh. 9. (peto or dersimonian or der simonian or fixed effect).tw,sh. 10. (retraction of publication or retracted publication).pt. 11. or/2-10 12. 1 and 11 13. meta-analysis.pt. 14. meta-analysis.sh. 15. (meta-analys$ or meta analys$ or metaanalys$).tw,sh. 16. (systematic$ adj5 review$).tw,sh. 17. (systematic$ adj5 overview$).tw,sh. 18. (quantitativ$ adj5 review$).tw,sh. 19. (quantitativ$ adj5 overview$).tw,sh. 20. (quantitativ$ adj5 synthesis$).tw,sh. 21. (methodologic$ adj5 review$).tw,sh. 22. (methodologic$ adj5 overview$).tw,sh. 23. (integrative research review$ or research integration).tw. 24. or/13-23 25. 12 or 24 |
| 1. Boolean operators | 1 AND 2 AND 3 |
| 1. Limits | Limit 4 to humans  Limit 5 to English language |
| **Cochrane Database of Systematic Reviews (7 articles returned)** | |
| 1. Exposure terms | (Nutrient* OR micronutrient* OR macronutrient* OR phytonutrient* OR antioxidant*) OR (vitamin* OR “vitamin D” OR “vitamin K” OR “vitamin C” OR “vitamin B*” OR thiamine OR riboflavin OR pyridoxine OR cyanocobalamin OR “dietary minerals” OR “iron intake” OR “zinc intake” OR “magnesium intake” OR “sodium intake” OR “potassium intake” OR “silicon intake” OR “boron intake” OR “calcium intake” OR “copper intake” OR “manganese intake” OR “iodine intake”) OR (“protein intake” OR “protein consumption” OR “animal protein” OR “plant protein” OR “dietary protein” OR “dietary fat*” OR “saturated fat” OR “mono-unsaturated fatty acids” OR “poly-unsaturated fatty acids” OR MUFAs OR PUFAs OR carbohydrate* OR sugar* OR “dietary fibre” OR “dietary fiber” OR “energy intake” OR “calorie intake” OR “caloric intake”) OR (“caffeinated intake” OR “caffeine intake” OR caffeine OR decaffeinated OR “decaffeinated intake” OR “dietary supplement*“ OR alcohol OR dairy OR milk OR cheese OR yogurt OR coffee OR meat OR fruit* OR vegetable* OR veg OR legumes OR nuts) OR (“dietary pattern” OR diet OR omnivore OR omnivorous OR vegetarian OR vegan OR plant-based OR “plant based” OR lacto-vegetarian OR lactovegetarian OR “Mediterranean diet” OR MedDiet OR “Western diet” OR “dietary approaches to stop hypertension” OR “DASH diet” OR fasting OR “dietary risk factor*”) OR [mh ^nutrients] OR [mh ^micronutrients] OR [mh ^phytochemicals] OR [mh ^antioxidants] OR [mh ^vitamins] OR [mh ^”vitamin D”] OR [mh ^”vitamin A deficiency”] OR [mh ^”vitamin B deficiency”] OR [mh ^”vitamin D deficiency”] OR [mh ^”vitamin E deficiency”] OR [mh ^”vitamin K deficiency”] OR [mh ^thiamine] OR [mh ^riboflavin] OR [mh ^pyridoxine] OR [mh ^”vitamin B12”] OR [mh ^”trace elements”] OR [mh ^”iron, dietary”] OR [mh ^”magnesium deficiency”] OR [mh ^”potassium deficiency”] OR [mh ^”sodium, dietary”] OR [mh ^”sodium chloride, dietary”] OR [mh ^”calcium, dietary”] OR [mh ^”Dietary proteins”] OR [mh ^”animal proteins, dietary”] OR [mh ^”fruit proteins”] OR [mh ^”grain proteins”] OR [mh ^”plant proteins, dietary”] OR [mh ^”dietary fats”] OR [mh ^butter] OR [mh ^”cholesterol, dietary”] OR [mh ^”dietary fats, unsaturated“] OR [mh ^margarine] OR [mh ^”fats, unsaturated”] OR [mh ^”dietary carbohydrates”] OR [mh ^”dietary fiber”] OR [mh ^”dietary sugars”] OR [mh ^starch] OR [mh ^”energy intake”] OR [mh ^”caloric restriction”] OR [mh ^”portion size”] OR [mh ^”serving size”] OR [mh ^Caffeine] OR [mh ^”dietary supplements”] OR [mh ^”alcohol abstinence”] OR [mh ^”alcohol drinking”] OR [mh ^”dairy products”] OR [mh ^butter] OR [mh ^”cultured milk products”] OR [mh ^”ice cream”] OR [mh ^margarine] OR [mh ^milk] OR [mh ^coffee] OR [mh ^meat] OR [mh ^”meat products”] OR [mh ^poultry] OR [mh ^”red meat”] OR [mh ^seafood] OR [mh ^fruit] OR [mh ^nuts] OR [mh ^seeds] OR [mh ^vegetables] OR [mh ^diet] OR [mh ^”diet, carbohydrate-restricted”] OR [mh ^”diet, fat-restricted“] OR [mh ^”diet, gluten-free”] OR [mh ^”diet, high-fat”] OR [mh ^”diet, high-protein”] OR [mh ^”diet, Mediterranean”] OR [mh ^”diet, vegetarian”] OR [mh ^”diet, western”] OR [mh ^”dietary approaches to stop hypertension”] OR [mh ^fasting] OR [mh ^vegetarians] OR [mh ^vegans] |
| 1. Primary outcome terms | (“Hip fracture” OR “osteoporotic fracture” OR “bone fracture” OR “fragility fracture”) OR [mh ^"hip fractures"] OR [mh ^"femoral neck fractures"] NOT treatment |
| 1. Boolean operators | 1 AND 2 |
| **Web of Science (387 articles returned)** | |
| 1. Exposure terms | TS=((Nutrient* OR micronutrient* OR macronutrient* OR phytonutrient* OR antioxidant*) OR (vitamin* OR “vitamin D” OR “vitamin K” OR “vitamin C” OR “vitamin B*” OR thiamine OR riboflavin OR pyridoxine OR cyanocobalamin OR “dietary minerals” OR “iron intake” OR “zinc intake” OR “magnesium intake” OR “sodium intake” OR “potassium intake” OR “silicon intake” OR “boron intake” OR “calcium intake” OR “copper intake” OR “manganese intake” OR “iodine intake”) OR (“protein intake” OR “protein consumption” OR “animal protein” OR “plant protein” OR “dietary protein” OR “dietary fat*” OR “saturated fat” OR “mono-unsaturated fatty acids” OR “poly-unsaturated fatty acids” OR MUFAs OR PUFAs OR carbohydrate* OR sugar* OR “dietary fibre” OR “dietary fiber” OR “energy intake” OR “calorie intake” OR “caloric intake”) OR (“caffeinated intake” OR “caffeine intake” OR caffeine OR decaffeinated OR “decaffeinated intake” OR “dietary supplement*“ OR alcohol OR dairy OR milk OR cheese OR yogurt OR coffee OR meat OR fruit* OR vegetable* OR veg OR legumes OR nuts) OR (“dietary pattern” OR diet OR omnivore OR omnivorous OR vegetarian OR vegan OR plant-based OR “plant based” OR lacto-vegetarian OR lactovegetarian OR “Mediterranean diet” OR MedDiet OR “Western diet” OR “dietary approaches to stop hypertension” OR “DASH diet” OR fasting OR “dietary risk factor*”)) |
| 1. Primary outcome terms | TS=(“Hip fracture” OR “osteoporotic fracture” OR “bone fracture” OR “fragility fracture”) NOT TS=treatment |
| 1. Review terms | TS=(“systematic review” OR meta-analysis OR metaanalysis OR “meta analysis”) |
| 1. Boolean operators | (1 AND 2 AND 3) AND LANGUAGE: (English) |

Each search is comprised of: 1) Dietary exposure terms AND 2) Outcome (hip fracture) terms AND 3) Review filter terms. Dietary exposure terms are either foods, nutrients, beverages, or dietary patterns. Black text = keywords; blue text = subject headings.
