# Supplementary material for: Dietary risk factors for hip fracture in adults: An umbrella review of meta-analyses of prospective cohort studies
Source: PLoS One. 2021 Nov 10;16(11):e0259144. doi: 10.1371/journal.pone.0259144 (PMC8580223; doi:10.1371/journal.pone.0259144)
Supplement: S2 Table — (DOCX) [file pone.0259144.s002.docx]

**S2 Table: Excluded articles with justifications for their exclusion.**

| Angelino et al. (2019) | Umbrella review |
| --- | --- |
| Avenell et al. (2014) | Supplements |
| Avenell et al. (2009) | Old version of a review |
| Avenell et al. (2005) | Old version of a review |
| Bailey et al. (2015) | No meta-analysis |
| Benetos et al. (2007) | No meta-analysis |
| Benetou et al. (2018) | Cohort pooling project |
| Benetou et al. (2016) | Cohort pooling project |
| Berg et al. (2008) | Included case-control studies |
| Bergholdt et al. (2018) | Cohort pooling project |
| Bischoff-Ferrari et al. (2005) | Supplements |
| Bischoff-Ferrari (2010) | No meta-analysis |
| Bjelakovic et al. (2014) | No relevant outcome |
| Bolanos and Francia (2010) | No relevant outcome |
| Bolland et al. (2014a) | Review of meta-analyses |
| Bolland et al. (2015) | Supplements |
| Bolland et al. (2018) | Supplements |
| Bolland et al. (2014b) | Full text not found |
| Bonjour et al. (2013) | No meta-analysis |
| Brown (2008) | Umbrella review |
| Cawood et al. (2012) | No relevant outcome |
| Ceylan et al. (2020) | No relevant outcome |
| Chakhtoura et al. (2020) | Umbrella review |
| Chen et al. (2014) | Included case-control studies. Subgroup analysis restricting to cohort studies was on fracture generally, not specified to hip. |
| Chung et al. (2011) | No relevant outcome |
| Darling et al. (2016) | Conference abstract |
| Darling et al. (2019) | No relevant outcome |
| Darling et al. (2017) | Old version of a review |
| de Macedo et al. (2017) | No meta-analysis |
| Dennehy and Tsourounis (2010) | Umbrella review |
| Dong et al. (2014) | HIV/hepatitis C virus coinfection patients |
| Drake et al. (2012) | Included case-control studies |
| Eleni and Panagiotis (2020) | Supplements |
| Fabiani et al. (2019) | Included case-control studies |
| Fang et al. (2020) | Included cross-sectional and case-control studies |
| Farsinejad-Marj et al. (2016) | Included case-control studies |
| Frost et al. (2013) | Conference abstract |
| Frost et al. (2016) | Conference abstract |
| Gaugris et al. (2005) | No meta-analysis |
| Gillespie et al. (2009) | No relevant outcome |
| Gillespie et al. (2000) | Old version of a review |
| Guo et al. (2017) | No relevant outcome |
| Hamishehkar et al. (2016) | No meta-analysis |
| Handoll et al. (2009) | No meta-analysis |
| Hao et al. (2017) | No relevant outcome |
| Hiligsmann et al. (2017) | No meta-analysis |
| Hill et al. (2018) | No relevant outcome |
| Ho-Pham et al. (2009) | No relevant outcome |
| Hoidrup et al. (2000) | Lack of dietary exposure (considered tobacco smoking as exposure) |
| Holvik et al. (2019) | Cohort pooling project |
| Huang et al. (2006) | No meta-analysis |
| Iwamoto et al. (2009) | No meta-analysis |
| Izaks (2007) | No relevant outcome |
| Jackson et al. (2007) | No relevant outcome |
| Jackson and Sheehan (2005) | No meta-analysis |
| Kahwati et al. (2018) | Supplements |
| Kanis et al. (2005a) | Cohort pooling project |
| Kanis et al. (2005b) | Cohort pooling project |
| Kanis et al. (2008) | No meta-analysis |
| Kunutsor et al. (2017) | Included case-control studies |
| Lai et al. (2010) | Supplements |
| Lee et al. (2014) | Included case-control studies |
| Li et al. (2020) | Supplements |
| Liu et al. (2012) | No relevant outcome |
| Lv et al. (2017) | Lack of dietary exposure (considered serum vitamin D levels) |
| MacLean et al. (2008) | Umbrella review |
| Man et al. (2016) | No relevant outcome |
| Mosekilde et al. (2007) | No meta-analysis |
| Mott et al. (2019) | No relevant outcome |
| Mozaffari et al. (2020) | No relevant outcome |
| Mozaffari et al. (2018) | Included case-control studies |
| Murad et al. (2012) | Included only trials whose populations had or were at risk of osteoporosis, had previous fractures, or other chronic diseases (patient population) |
| Nakamura and Masayuki (2006) | Umbrella review |
| Oliver et al. (2007) | Umbrella review |
| Orchard et al. (2012) | No relevant outcome |
| Ong et al. (2018) | No meta-analysis |
| Papadimitriou et al. (2017) | Cohort pooling project |
| Pedersen and Cederholm (2014) | No meta-analysis |
| Peraza-Delgado et al. (2020) | Umbrella review |
| Plawecki and Chapman-Novakofski (2010) | No meta-analysis |
| Pripp and Dahl (2015) | Umbrella review |
| Ruan et al. (2015) | No relevant outcome |
| Sawka et al. (2010) | No meta-analysis |
| Scragg (2012) | Umbrella review |
| Shams-White et al. (2017) | No relevant outcome |
| Shams-White et al. (2018) | No relevant outcome |
| Shen et al. (2015) | Lack of dietary exposure (considered cigarette smoking) |
| Shi et al. (2019) | Lack of dietary exposure (considered depression) |
| Solbakken et al. (2014) | Not a systematic review |
| Sun et al. (2018) | Included case-control studies |
| Tang et al. (2007) | Supplements |
| Theodoratou et al. (2014) | Umbrella review |
| Thorning et al. (2016) | Umbrella review |
| Trakanoska et al. (2018) | Not a systematic review |
| van den Heuvel and Steijns (2018) | Narrative review |
| van der Velde et al. (2014) | Umbrella review |
| Veronese et al. (2015) | Lack of dietary exposure (considered vitamin K antagonists use) |
| Vestergaard et al. (2003) | Lack of dietary exposure (considered smoking) |
| Wallace et al. (2020) | No meta-analysis |
| Wang et al. (2020) | Lack of dietary exposure (considered serum vitamin D levels) |
| Weatherall (2000) | Lack of dietary exposure (considered serum vitamin D levels) |
| Weaver et al. (2016) | Supplements |
| Wikoff et al. (2017) | No relevant outcome |
| Wu et al. (2019) | Population was restricted to knee osteoarthritis patients (too narrow) |
| Wu et al. (2016) | Lack of dietary exposure (considered cigarette smoking) |
| Xiang et al. (2019) | No meta-analysis of cohort studies for risk of hip fracture (included CCs and other sites, inseparable) |
| Xu et al. (2017) | Not a systematic review |
| Yan et al. (2015) | Included case-control studies, and subgroup analysis restricting to just cohort studies was only available for total fracture, not hip fracture |
| Yang et al. (2012) | Lack of dietary exposure (considered serum homocysteine levels) |
| Yao et al (2019) | Supplements |

Reasons stated for exclusion are the primary reasons; studies may have multiple reasons for exclusion.
