# Supplementary material for: Dietary risk factors for hip fracture in adults: An umbrella review of meta-analyses of prospective cohort studies
Source: PLoS One. 2021 Nov 10;16(11):e0259144. doi: 10.1371/journal.pone.0259144 (PMC8580223; doi:10.1371/journal.pone.0259144)
Supplement: S3 Table — (DOCX) [file pone.0259144.s003.docx]

**S3 Table: Characteristics of eligible meta-analyses assessing the association between risk of hip fracture and dietary exposures.**

| **Exposure** | **Author (year)** | **Population** | **n cohorts** | **Follow-up range (years)** | **n subjects** | **n events** | **Exposure ascertainment** | **Outcome ascertainment** | **Comparison** | **Summary effect estimate (95% CI)** | **I^2^ (%)** | **Egger's p-value** | **Fully adjusted confounders** |
| --- | --- | --- | --- | --- | --- | --- | --- | --- | --- | --- | --- | --- | --- |
| **Dietary patterns** | | | | | | | | | | | | | |
| Alternative healthy eating index | Panahande et al. (2018) | Adults (30 - 79 y) | 4 | 10 - 32 | 264319 | 6212 | FFQ | Self-reported questionnaires, medical records | High vs low | RR: 0.83 (0.71, 0.97) | N/A | 0.189 | Sex |
| Mediterranean diet | Malmir et al. (2018a) | Adults (> 35 y) | 4 | 8-16 | 351625 | 6253 | FFQ, diet history questionnaire, 24 h recall | N/A | Per 1 unit increase in MD score | RR: 0.95 (0.92, 0.98) | 68 | 0.78 | Age, sex, BMI, smoking |
| **Food groups** | | | | | | | | | | | | | |
| Dairy | Bian et al. (2018) | Adults (> 30 y) | 10 | 3-22 | 363557 | 8613 | FFQ (validated, non-validated), or N/A | Self-reported, hospital registers, medical records, radiographic reports | High vs low | RR: 0.87 (0.76, 1.00) | 81* | N/A | None |
| Dairy | Malmir et al. (2019) | Adults (> 18 y) | 5 | 4-21 | 189501 | 9091 | FFQ | Self-reported, hospital registers | Per 200 g increase/day | RR: 0.98 (0.95, 1.01) | N/A | 0.223 | None |
| Dairy | Matia-martin et al. (2019) | Healthy non-Hispanic whites (> 26 y) | 4 | 8-22 | 231442 | 8629 | FFQ (self-reported, reviewed with clinic staff) | Self-reported, small validation study, medical records, hospital registers, radiographic reports | Per 'increment' increase | RR: 0.98 (0.95, 1.01) | 86* | 0.982 | Age, sex, smoking, calcium and vitamin D supplementation, total energy intake |
| Milk | Bian et al. (2018) | Adults (> 30 y) | 7 | 7-22 | 301590 | 7868 | FFQ (validated, non-validated), or N/A | Self-reported, hospital registers, medical records, radiographic reports | Per 200 g increase/day | RR: 1.00 (0.94, 1.07) | 87* | 0.81 | Age |
| Milk | Bischoff-Ferrari et al. (2011) | Middle-aged or older men | 3 | 8-14 | 75149 | 195 | FFQ | N/A | Per 300 mg increase/day | RR: 0.91 (0.81, 1.01) | N/A | N/A | Age, sex |
|  |  | Middle-aged or older women | 6 | 3-26 | 195102 | 3574 | FFQ | N/A | Per 300 mg increase/day | RR: 0.99 (0.96, 1.02) | N/A | N/A | Age, sex |
| Milk | Hidayat et al. (2020) | Adults (mean age > 50 y) | 7 | 6-22 | 383122 | 15020 | FFQ | Self-reported, medical records, radiographic reports, hospital registers | Per 1 glass increase/day | RR: 0.97 (0.92, 1.03) | N/A | 0.21 | Age, sex |
| Milk | Malmir et al. (2019) | Adults (> 18 y) | 8 | 4-50 | 348181 | 33446 | FFQ | Self-reported, hospital records, x-ray exam | Per 200 g increase/day | RR: 1.09 (1.07, 1.11) | N/A | 0.015 | None |
| Milk | Matia-martin et al. (2019) | Adults (> 26 y) | 5 | 8-22 | 236136 | 8454 | FFQ (self-reported, reviewed with clinic staff) | Self-reported, small validation study, medical records, hospital registers, radiographic reports | Per 'increment' increase | RR: 1.01 (0.96, 1.06) | 84 | N/A | Age, sex, smoking, calcium and vitamin D supplementation, total energy intake |
| Yogurt | Bian et al. (2018) | Adults (> 30 y) | 3 | 8-20 | 109018 | 5579 | FFQ (validated, non-validated), or N/A | Self-reported, hospital registers, medical records, radiographic reports | High vs low | RR: 0.75 (0.66, 0.86) | 0 | N/A | Age, sex, calcium and vitamin D supplement use |
| Yogurt | Hidayat et al. (2020) | Adults (mean > 50 y) | 4 | 12-21 | 234654 | 8217 | FFQ | Self-reported, medical records, radiographic reports, hospital registers | High vs low | RR: 0.78 (0.68, 0.90) | 14 | >0.45 | Age, sex, height, smoking, calcium and vitamin D supplementation, total energy intake |
| Yogurt | Matia-martin et al. (2019) | Adults (> 26 y) | 5 | 8-22 | 236136 | 8454 | FFQ (self-reported, reviewed with clinic staff) | Self-reported, small validation study, medical records, hospital registers, radiographic reports | Per 'increment' increase | RR: 0.96 (0.91, 1.01) | 72* | N/A | Age, sex, smoking, calcium and vitamin D supplementation, total energy intake |
| Yogurt | Ong et al. (2020) | Postmenopausal women (age > 55 y) | 3 | 12-22 | 108219 | 6991 | FFQ | N/A | High vs low | RR: 0.76 (0.63, 0.92) | 29 | N/A | Age, sex, BMI, height, smoking |
| Cheese | Bian et al. (2018) | Adults (> 30 y) | 3 | 7-20 | 117240 | 5648 | FFQ (validated, non-validated), or N/A | Self-reported, registers, medical records, radiographic and operative reports | High vs low | RR: 0.68 (0.61, 0.77) | 0 | N/A | Age, sex, calcium and vitamin D supplement use |
| Cheese | Hidayat et al. (2020) | Adults (mean> 50 y) | 4 | 6-21 | 305157 | 8860 | FFQ | Self-reported, medical records, radiographic reports, hospital registers | High vs low | RR: 0.85 (0.66, 1.08) | 77* | p>0.45 | Age, sex, smoking |
| Cheese | Matia-martin et al. (2019) | Adults (> 26 y) | 4 | 8-21 | 232924 | 8411 | FFQ (self-reported, reviewed with clinic staff) | Self-reported, small validation study, medical records, hospital registers, radiographic reports | Per 'increment' increase | RR: 0.96 (0.88, 1.04) | 91* | N/A | Age, sex, smoking, calcium and vitamin D supplementation, total energy intake |
| Cheese | Ong et al. (2020) | Postmenopausal women (age > 55 y) | 2 | 12-22 | 81069 | 2214 | FFQ (self-reported, reviewed with clinic staff) | Self-reported, small validation study, medical records, hospital registers, radiographic reports | High vs low | RR: 0.89 (0.73, 1.10) | 0 | N/A | Age, sex, BMI, height, smoking |
| Fruits | Luo et al. (2016) | Middle-aged to older adults (37 - 95 y) | 5 | 8-14 | 329125 | 6133 | FFQ | Telephone interviews, self-reported questionnaire, hospital registers | High vs low | HR: 0.91 (0.77, 1.07) | 73* | N/A | Age, sex, alcohol intake, total energy intake physical activity, smoking. |
| Vegetables | Luo et al. (2016) | Middle-aged to older adults (37 - 95 y) | 5 | 8-14 | 329125 | 6133 | FFQ | Telephone interviews, self-reported questionnaire, hospital registers | High vs low | HR: 0.81 (0.68, 0.96) | 71* | N/A | Age, sex, alcohol intake, total energy intake physical activity, smoking. |
| Fruits and vegetables | Luo et al. (2016) | Middle-aged to older adults (37 - 95 y) | 7 | 8-14 | 329125 | 6133 | FFQ | Telephone interviews, self-reported questionnaire, hospital registers | High vs low | HR: 0.88 (0.78, 1.01) | 74* | N/A | Age, sex, alcohol intake, total energy intake physical activity, smoking. |
| Fruits and vegetables | Brondani et al. (2019) | Adults (age > 50 y) | 5 | 7-20 | 218926 | N/A | Validated FFQ | N/A | High vs low | RR: 0.92 (0.87, 0.98) | 56 | 0.147 | BMI, calcium and vitamin D supplementation |
| Tea | Sheng et al. (2013) | Adults | 3 | 6-12 | 136413 | 5171 | FFQ | Medical records, self-reported, hospital registers | High vs low | RR: 1.03 (0.54, 1.52) | 42 | 0.06 | Age, sex, alcohol intake, HRT |
| Coffee | Li et al. (2015) | Adults (> 30 y) | 9 | 4-30 | 205930 | 5408 | FFQ | Radiographic reports, hospital registers, self-reported, medical records | High vs low | RR: 1.13 (0.86, 1.48) | 79* | 0.181 | Sex |
| Coffee | Li and Xu (2013) | Adults (> 34 y) | 4 | 6-30 | 138009 | 857 | FFQ | Hospital registers, medical records, telephone, self-report | Per cup increase/day | OR: 1.00 (0.96, 1.03) | N/A | 0.891 | Age, sex |
| Coffee | Sheng et al. (2013) | Adults | 6 | 6-30 | 184947 | 5888 | FFQ | Medical records, self-reported, hospital registers | High vs low | RR: 1.09 (0.60, 1.58) | 68* | < 0.01 | Sex |
| Total alcohol | Zhang et al. (2014) | Adults (> 20 y) | 18 | 3-30 | 3730424 | 26168 | Self-administered FFQ | Self-reported, radiographic report, medical records | Any vs none | RR: 1.03 (0.91, 1.15) | 72* | > 0.1 | Age, sex |
|  |  |  | 7 |  | 3363045 | 22072 |  |  | Light (0.01 - 12.5 g/d) vs none | RR: 0.88 (0.83, 0.92) | 20 | > 0.1 | Age, sex |
|  |  |  | 7 |  | 3337647 | 21704 |  |  | Moderate (12.6 - 49.9 g/d) vs none | RR: 1.00 (0.85, 1.14) | 56* | > 0.1 | Age, sex |
|  |  |  | 3 |  | 3363253 | 21637 |  |  | Heavy (> 50 g/d) vs none | RR: 1.71 (1.41, 2.01) | 0 | > 0.1 | Age, sex, smoking, BMI |
| Wine | Zhang et al. (2014) | Adults (> 20 y) | 4 | 3-14 | 237789 | 2574 |  |  | Any vs no alcohol | RR: 0.81 (0.71, 0.92) | 0 | > 0.1 | Age, sex |
| Beer | Zhang et al. (2014) | Adults (> 20 y) | 4 | 3-14 | 237789 | 2574 |  |  | Any vs no alcohol | RR: 1.13 (0.69, 1.56) | 79* | > 0.1 | Age, sex |
| Liquor | Zhang et al. (2014) | Adults (> 20 y) | 4 | 3-14 | 237789 | 2574 |  |  | Any vs no alcohol | RR: 0.94 (0.75, 1.12) | 33 | > 0.1 | Age, sex |
| **Macronutrients** | | | | | | | | | | | | | |
| Protein | Darling et al. (2009) | Adults (35 – 74 y) | 3 | 1-22 | 120829 | N/A | FFQ, National survey data | Self-reported, medical records | High vs low | RR: 0.75 (0.47, 1.20) | 20 | N/A | Age, sex, weight, BMI, physical activity, menopausal status, smoking, HRT, alcohol, calcium intake |
| Protein | Groenendijk et al. (2019) | Older men and women (> 65 y) | 4 | 6-32 | 152779 | N/A | FFQ, biomarkers | N/A | High vs low | RR: 0.89 (0.84, 0.94) | 0 | N/A | Sex |
| Protein | Wu et al. (2015) | Adults (> 18 y) | 6 | N/A | 270011 | 3787 | N/A | N/A | High vs low | RR: 0.89 (0.82, 0.97) | 0 | 0.054 | Sex |
| Animal protein | Wu et al. (2015) | Adults (> 18 y) | 4 | N/A | 161393 | 535 | N/A | N/A | High vs low | RR: 1.04 (0.70, 1.54) | 52 | N/A | Age, sex, smoking, physical activity |
| Animal protein | Darling et al. (2009) | Middle-aged or older adults | 3 | 1-22 | 157737 | N/A | FFQ | Self-reported, medical records | High vs low | RR: 0.83 (0.64, 1.30) | 48 | N/A | Age, sex, weight, BMI, physical activity, menopausal status, smoking, HRT use, alcohol, calcium intake |
| Vegetable protein | Wu et al. (2015) | Adults (> 18 y) | 3 | N/A | 121606 | 322 | N/A | N/A | High vs low | RR: 1.00 (0.53, 1.91) | 57 | N/A | Age, sex, smoking, physical activity |
| Vegetable protein | Darling et al. (2009) | Women (35 – 69 y) | 2 | 1-12 | 117950 | N/A | FFQ | Self-reported, medical records | High vs low | RR: 1.21 (0.82, 1.79) | 2 | N/A | Age, sex, weight, BMI, physical activity, menopausal status, smoking, HRT, alcohol, calcium intake |
| **Micronutrients** | | | | | | | | | | | | | |
| Dietary calcium | Bischoff-Ferrari et al. (2007) | Middle-aged or older women | 4 | 3-18 | 83198 | 854 | 24 h recall, FFQ | N/A | Per 300 mg increase/day | RR: 1.01 (0.96, 1.06) | N/A | N/A | Sex |
| Dietary calcium | Cumming and Nevitt (1997) | Postmenopausal women (> 50 y) | 5 | 4-15 | 28511 | 915 | 24 h recall, FFQ | N/A | Per 300 mg increase/day | OR: 0.96 (0.91, 1.02) | N/A | N/A | Sex, measurement error |
| Dietary calcium | Wang et al. (2015) | Adults (> 34 y) | 8 | 7-18 | 267762 | 2435 | 24 h recall, FFQ | Postcard or telephone interview, medical record | High vs low | RR: 0.97 (0.88, 1.06) | 0 | 0.06 | Age, sex |
| Dietary calcium | Xu et al. (2007) | Women (> 35 y) | 5 | 5-15 | 41645 | 941 | 24 h recall, FFQ | N/A | High vs low | RR: 0.96 (0.89, 1.04) | N/A | 0.54 | Age, sex |
| Dietary vitamin C | Malmir et al. (2018b) | Adults (39-80 y) | 3 | 13-15 | 6282 | 584 | 24 h recall, FFQ, 7-day food record | Interview, hospital record, medical report, or N/A | High vs low | RR: 0.92 (0.59, 1.44) | 55 | 0.83 | Age, sex |
| Dietary vitamin C | Zeng et al. (2020) | Adults (39-80 y) | 2 | 13-15 | N/A | N/A | 7-day food record or FFQ | N/A | High vs low | RR: 0.92 (0.59, 1.44) | 55 | N/A | Age, sex, total energy intake, HRT, BMI |
| Dietary vitamin A | Wu et al. (2014) | Adults (20-95 y) | 3 | N/A | 182787 | 1716 | N/A | N/A | High vs low | RR: 1.29 (1.07, 1.57) | 0 | 0.312 | Age, sex, total energy intake, alcohol, BMI |
| Dietary vitamin A | Zhang et al. (2017) | Adults (> 34 y) | 3 | 12-18 | 200716 | 1716 | FFQ | Self-reported, medical records | High vs low | RR: 1.29 (1.06, 1.57) | 0 | 0.85 | Age, sex, BMI, alcohol |
| Dietary carotenoids | Xu et al. (2017) | Adults | 2 | 10-17 | 62470 | 1730 | FFQ | Medical records, radiographic reports | High vs low | OR: 0.72 (0.51, 1.01) | 59 | 0.16 | Age, sex, BMI, total energy intake, smoking, physical activity, calcium intake |
| Dietary retinol | Wu et al. (2014) | Adults (20-95 y) | 4 | N/A | 183907 | 1963 | N/A | N/A | High vs low | RR: 1.40 (1.03, 1.91) | 64* | 0.338 | Total energy intake, sex, BMI |
| Dietary retinol | Zhang et al. (2017) | Adults (> 34 y) | 4 | 3-18 | 201836 | 1963 | FFQ | Self-reported, medical records | High vs low | RR: 1.40 (1.02, 1.91) | 65* | 0.17 | Sex, BMI, medicine use |
| Dietary a-carotene | Xu et al. (2017) | Adults (> 56 y) | 2 | 10-17 | 62470 | 1730 | FFQ | Medical records, radiographic reports | High vs low | OR: 0.77 (0.55, 1.08) | 64* | 0.36 | Age, sex, BMI, total energy intake, smoking, physical activity, calcium |
| Dietary b-carotene | Wu et al. (2014) | Adults (20-95 y) | 3 | N/A | 136523 | 2333 | N/A | N/A | High vs low | RR: 0.82 (0.59, 1.14) | 78* | 0.406 | Age, sex, BMI, smoking, physical activity, calcium, HRT |
| Dietary b-carotene | Xu et al. (2017) | Adults (mean > 56 y) | 3 | 10-17 | 134807 | 2333 | FFQ | Medical records, radiographic reports | High vs low | OR: 0.84 (0.62, 1.14) | N/A | 0.1 | Age, sex, BMI, smoking, physical activity, calcium |
| Dietary b-carotene | Zhang et al. (2017) | Adults (> 34 y) | 2 | 17-18 | 135594 | 2233 | FFQ | Self-reported, medical records | High vs low | RR: 0.91 (0.64, 1.31) | 82* | 0.8 | Age, sex, BMI, calcium intake, smoking, physical activity, HRT |
| Dietary b-cryptoxanthin | Xu et al. (2017) | Adults (mean > 56 y) | 2 | 10-17 | 62470 | 1730 | FFQ | Medical records, radiographic reports | High vs low | OR: 1.11 (0.97, 1.28) | 0 | 0.49 | Age, sex, BMI, total energy intake, smoking, physical activity, calcium |
| Dietary lycopene | Xu et al. (2017) | Adults (mean > 56 y) | 2 | 10-17 | 62470 | 1730 | FFQ | Medical records, radiographic reports | High vs low | OR: 0.84 (0.69, 1.01) | 8 | 0.14 | Age, sex, BMI, total energy intake, smoking, physical activity, calcium |
| Dietary lutein/zeaxanthin | Xu et al. (2017) | Adults (mean > 56 y) | 2 | 10-17 | 62470 | 1730 | FFQ | Medical records, radiographic reports | High vs low | OR: 0.94 (0.79, 1.11) | 8 | 0.6 | Age, sex, BMI, total energy intake, smoking, physical activity, calcium |
| Dietary ALA | Sadheghi et al. (2019) | Adults (> 30 y) | 3 | 8-24 | 260106 | 3316 | FFQ (self-reported or interview) | Blinded clinician using medical records, self-reported, interview | High vs low | RR: 1.01 (0.90, 1.13) | 71* | N/A | Sex, total energy intake, BMI |
| Dietary EPA + DHA | Sadheghi et al. (2019) | Adults (> 30 y) | 4 | 8-24 | 265151 | 3821 | FFQ (self-reported or interview) | Blinded clinician using medical records, self-reported, interview | High vs low | RR: 0.91 (0.81, 1.03) | 0 | N/A | Sex, total energy intake, BMI |
| Antioxidant vitamin intake | Zhou et al. (2020) | Adults (28-95 y) | 9 | 4-19 | 329531 | 9052 | N/A | N/A | High vs low | RR: 0.87 (0.69, 1.08) | 89* | 0.447 | Age, sex, BMI |

N/A = not applicable or available; * = significant heterogeneity; RR = relative risk; OR = odds ratio; HR = hazard ratio; 95% CI = 95% confidence interval. For milk and coffee consumption, I^2^ and Egger’s p-value were unobtainable from dose-response meta-analyses, thus values from high vs low comparisons are presented as an estimate where available. For antioxidant vitamin intake, Egger’s p value was only available for total fracture. Adjustment for confounders includes only those factors that were adjusted for in all of a meta-analyses’ included cohort studies. Where meta-analyses included only single-sex studies or studies that adjusted for sex and single-sex studies, sex was considered fully adjusted for. BMI = body mass index; HRT = hormone replacement therapy, including oestrogen use. y = years.
