# Supplementary material for: Dietary risk factors for hip fracture in adults: An umbrella review of meta-analyses of prospective cohort studies
Source: PLoS One. 2021 Nov 10;16(11):e0259144. doi: 10.1371/journal.pone.0259144 (PMC8580223; doi:10.1371/journal.pone.0259144)
Supplement: S4 Table — (DOCX) [file pone.0259144.s004.docx]

**S4 Table: Source of funding for eligible systematic reviews.**

| **Author (year)** | **Source of Funding** |
| --- | --- |
| Bian et al. (2018) | National Natural Science Foundation of China |
| Bischoff-Ferrari et al. (2007) | Medical Foundation (Charles H Farnsworth Trust; US Trust Company; Trustee and the Charles A King Trust; Fleet National Bank) and the International Foundation for the Promotion of Nutrition Research and Nutrition Education (ISFE); the Swiss Foundation for Nutrition Research (SFEFS), and the Swiss National Foundation (SNF Professorship grant) |
| Bischoff-Ferrari et al. (2011) | Vontobel Foundation, The Baugarten Foundation, a Swiss National Foundations Professorship Grant (PP00B‐114864), and the Velux Foundation. |
| Brondani et al. (2019) | The Coordination of Higher-Level Personnel (CAPES), Brazil; The Federal University of Santa Maria (UFSM) |
| Cumming and Nevitt (1997) | N/A |
| Darling et al. (2009) | None |
| Groenendijk et al. (2019) | Jaap Schouten Foundation |
| Hidayat et al. (2020) | National Key R&D Program of China (No. 2017YFC1310700, No. 2017YFC1310701) and the Suzhou Science and Technology Bureau (No. SYS201741). |
| Li and Xu (2013) | National Natural Science Foundation of China and the Universities Natural Science Foundation of Jiangsu Province |
| Li et al. (2015) | None |
| Luo et al. (2016) | N/A |
| Malmir et al. (2018a) | Joint collaboration of Endocrinology and Metabolism Molecular-Cellular Sciences Institute, Tehran University of Medical Sciences, and School of Nutritional Sciences and Dietetics, Tehran University of Medical Sciences, Tehran, Iran, and the Iran National Science Foundation (INSF) |
| Malmir et al. (2018b) | N/A |
| Malmir et al. (2019) | N/A |
| Matia-Martin et al. (2019) | Interprofessional Dairy Organization (INLAC), Spain, GenObIA-CM with reference (S2017/BMD-3773), the Comunidad de Madrid and cofinanced with Structural Funds of the European Union; from Instituto de Salud Carlos III supported with funds from the Spanish Ministry of Health and FEDER (PI17/1732); and from Fundación de Investigación en Nutrición y Metabolismo (FINUMET). |
| Ong et al. (2020) | None |
| Panahande et al. (2018) | Students’ Scientific Research Center, Tehran University of Medical Sciences, Tehran, Iran |
| Sadeghi et al. (2019) | Not reported |
| Sheng et al. (2013) | Fund for Key National Basic Research Program of China (grant 2012CB619101), Major Basic Research of Science and Technology Commission of Shanghai Municipality (grant no. 11DJ1400303), Key Disciplines of Shanghai Municipal Education Commission (grant no. J50206), Scientific Research from the National Natural Science Foundation for the Youth of China (grant no. 81201364), and Innovative Research from Shanghai Municipal Education Commission (grant no. 13YZ031) |
| Wang et al. (2015) | National Natural Science Foundation of China |
| Wu et al. (2014) | National Natural Science Foundation of China (81372014, 81371988); Department of Health of Zhejiang Province, Backbone of Talent Project (2012RCB037); and Department of Science and Technology of Wenzhou, Wenzhou Science and Technology Project (Y20120073) |
| Wu et al. (2015) | National Natural Science Foundation of China (81372014, 81371988); and Xinmiao talent plan of Zhejiang Province (2014R413053) |
| Xu et al. (2017) | National Natural Science Foundation of China [No_81372980, 81673150, 81001185] and the Priority Academic Program Development of Jiangsu Higher Education Institutions (PAPD) |
| Xu et al. (2007) | N/A |
| Zeng et al. (2020) | China Postdoctoral Science Foundation (No. 2018M633036), the Medical Science Research Foundation of Guangdong Province (No. B2019091), the National Natural Science Foundation of China (No. 81873314), the Project of Guangdong Provincial Department of Finance (Nos. [2014]157, [2018]8), Key scientific research platforms and research projects of universities in Guangdong Province (No. 2018KQNCX041), and the Science and Technology Research Project of Guangdong Provincial Hospital of Chinese Medicine (Nos. YN2019ML08, YK2013B2N19, and YN2015MS15) |
| Zhang et al. (2014) | Fund for Key National Basic Research Program of China (grant no. 2012CB619101 and 81401852), Natural Science Foundation of Science and Technology Commission of Shanghai Municipality (14ZR1424000), Major Basic Research of Science and Technology Commission of Shanghai Municipality (grant no. 11DJ1400303) |
| Zhang et al. (2017) | National Natural Science Foundation of China (Grant No. 81402668) |
| Zhou et al. (2020) | Jiangsu University Clinical Medicine Science and Technology Development Fund Project |

N/A = not applicable or available.
