# Supplementary material for: Dietary risk factors for hip fracture in adults: An umbrella review of meta-analyses of prospective cohort studies
Source: PLoS One. 2021 Nov 10;16(11):e0259144. doi: 10.1371/journal.pone.0259144 (PMC8580223; doi:10.1371/journal.pone.0259144)
Supplement: S5 Table — (DOCX) [file pone.0259144.s005.docx]

**S5 Table:** **Methodological quality assessments of eligible systematic reviews using the AMSTAR-2 tool.**

|  | **Domain** | | | | | | | | | | | | | | | | |
| --- | --- | --- | --- | --- | --- | --- | --- | --- | --- | --- | --- | --- | --- | --- | --- | --- | --- |
| **Study** | **1** | **2^c^** | **3** | **4^c^** | **5** | **6** | **7^c^** | **8** | **9^c^** | **10** | **11^c^** | **12** | **13^c^** | **14** | **15^c^** | **16** | **Score** |
| Bian et al. (2018) | 0 | 0 | 0 | 0.5 | 1 | 1 | 0 | 1 | 0.5 | 0 | 0 | 1 | 1 | 1 | 1 | 1 | Critically low |
| Bischoff-Ferrari et al. (2007) | 0 | 0 | 1 | 1 | 0 | 1 | 0 | 1 | 0 | 0 | 1 | 1 | 1 | 1 | 1 | 1 | Critically low |
| Bischoff-Ferrari et al. (2010) | 0 | 0 | 1 | 1 | 0 | 1 | 0 | 0 | 0 | 0 | 1 | 0 | 0 | 1 | 1 | 1 | Critically low |
| Brondani et al. (2019) | 1 | 1 | 1 | 0.5 | 1 | 1 | 0 | 0.5 | 0.5 | 0 | 0 | 0 | 1 | 1 | 1 | 1 | Critically low |
| Cumming and Nevitt (1997) | 0 | 0 | 1 | 0 | 0 | 0 | 0 | 1 | 0 | 0 | 1 | 0 | 0 | 1 | 0 | 0 | Critically low |
| Darling et al. (2009) | 0 | 0 | 0 | 0.5 | 1 | 0 | 0 | 1 | 0 | 0 | 0 | 0 | 1 | 1 | 0 | 1 | Critically low |
| Groenendijk et al. (2019) | 1 | 0 | 0 | 0.5 | 1 | 1 | 0 | 0 | 0.5 | 0 | 0 | 0 | 1 | 1 | 0 | 1 | Critically low |
| Hidayat et al. (2020) | 1 | 0 | 1 | 0.5 | 1 | 1 | 1 | 1 | 0.5 | 0 | 1 | 1 | 1 | 1 | 1 | 1 | Low |
| Li et al. (2015) | 0 | 0 | 1 | 0 | 1 | 1 | 0 | 1 | 0 | 0 | 1 | 0 | 0 | 1 | 1 | 1 | Critically low |
| Li and Xu (2013) | 0 | 0 | 0 | 0 | 1 | 1 | 1 | 1 | 0 | 0 | 1 | 0 | 1 | 1 | 1 | 1 | Critically low |
| Luo et al. (2016) | 0 | 0 | 0 | 0.5 | 1 | 1 | 1 | 0.5 | 0.5^b^ | 0 | 0 | 0 | 1 | 1 | 0 | 1 | Critically low |
| Malmir et al. (2020) | 1 | 0 | 0 | 0.5 | 1 | 0 | 0 | 1 | 0.5 | 0 | 1 | 1 | 1 | 1 | 1 | 1 | Critically low |
| Malmir et al. (2018a) | 1 | 0 | 0 | 0.5 | 1 | 0 | 1 | 1 | 0.5^a^ | 0 | 1 | 0 | 1 | 1 | 1 | 1 | Critically low |
| Malmir et al. (2018b) | 0 | 1 | 0 | 0.5 | 1 | 0 | 1 | 1 | 0.5^a^ | 0 | 0 | 0 | 1 | 1 | 1 | 1 | Critically low |
| Matia-Martin et al. (2019) | 0 | 1 | 0 | 0.5 | 1 | 1 | 0 | 1 | 0.5 | 0 | 1 | 0 | 0 | 0 | 1 | 1 | Critically low |
| Ong et al. (2019) | 1 | 1 | 0 | 0.5 | 1 | 1 | 0 | 1 | 0.5 | 0 | 0 | 0 | 1 | 1 | 0 | 1 | Critically low |
| Panahande et al. (2018) | 1 | 0 | 0 | 0.5 | 1 | 1 | 0 | 1 | 0.5^a^ | 0 | 1 | 0 | 1 | 0 | 1 | 1 | Critically low |
| Sadeghi et al. (2019) | 0 | 0 | 0 | 0.5 | 1 | 1 | 0 | 1 | 0.5^a^ | 0 | 0 | 0 | 1 | 1 | 1 | 1 | Critically low |
| Sheng et al. (2013) | 0 | 0 | 0 | 0 | 1 | 1 | 0 | 1 | 0 | 0 | 0 | 0 | 0 | 1 | 1 | 1 | Critically low |
| Wang et al. (2015) | 0 | 0 | 1 | 0 | 1 | 1 | 0 | 0 | 0 | 0 | 0 | 0 | 1 | 1 | 1 | 1 | Critically low |
| Wu et al. (2014) | 0 | 0 | 0 | 0.5 | 1 | 1 | 0 | 0 | 0.5 | 0 | 0 | 0 | 0 | 0 | 1 | 1 | Critically low |
| Wu et al. (2015) | 0 | 0 | 0 | 0.5 | 1 | 1 | 0 | 0 | 0.5 | 1 | 0 | 0 | 0 | 1 | 1 | 1 | Critically low |
| Xu et al. (2017) | 0 | 0 | 0 | 0.5 | 0 | 1 | 0 | 1 | 0.5^a^ | 0 | 0 | 0 | 1 | 0 | 1 | 1 | Critically low |
| Xu et al. (2007) | 1 | 0 | 1 | 0 | 1 | 1 | 0 | 0 | 0.5 | 0 | 1 | 1 | 1 | 0 | 1 | 0 | Critically low |
| Zeng et al. (2020) | 0 | 0 | 0 | 0.5 | 1 | 1 | 0 | 0 | 0.5^a^ | 0 | 0 | 0 | 1 | 1 | 1 | 1 | Critically low |
| Zhang et al. (2015) | 1 | 0 | 0 | 0 | 1 | 1 | 1 | 0 | 0 | 0 | 1 | 0 | 1 | 1 | 1 | 1 | Critically low |
| Zhang et al. (2017) | 0 | 0 | 0 | 0.5 | 1 | 1 | 0 | 1 | 0.5^a^ | 0 | 0 | 0 | 1 | 0 | 1 | 1 | Critically low |
| Zhou et al. (2020) | 0 | 0 | 1 | 0.5 | 1 | 1 | 0 | 0 | 0.5^a^ | 0 | 0 | 0 | 1 | 0 | 1 | 1 | Critically low |

^a^ = 0.5 was considered a critical flaw because risk of bias assessments per domain were not presented in the review. **^c^** = Critical domains. For item 11, dose-response meta-analyses or categorical comparisons using consistent thresholds were awarded a score of 1, whereas categorical comparisons without consistent thresholds (or with unclear threshold definitions) for defining exposure categories scored 0.
