# Supplementary material for: Dietary risk factors for hip fracture in adults: An umbrella review of meta-analyses of prospective cohort studies
Source: PLoS One. 2021 Nov 10;16(11):e0259144. doi: 10.1371/journal.pone.0259144 (PMC8580223; doi:10.1371/journal.pone.0259144)
Supplement: S6 Table — (DOCX) [file pone.0259144.s006.docx]

**S6 Table: Quality of evidence assessments per association from the highest quality meta-analysis per exposure using the GRADE tool.**

| **Exposure** | **Author (year)** | **Comparison** | **Number of studies** | **Study design** | **Risk of bias** | **Inconsistency** | **Indirectness** | **Imprecision** | **Other considerations** | **Overall quality** |
| --- | --- | --- | --- | --- | --- | --- | --- | --- | --- | --- |
| **Dietary patterns** | | | | | | | | | | |
| MD | Malmir et al. (2018a) | Increase of 1 in adherence score | 4 | Prospective cohort | Serious^c^ | Not serious | Not serious | Not serious | None | ⨁◯◯◯ |
| AHEI | Panahande et al. (2018) | High vs low | 4 | Prospective cohort | Serious^c^ | Not serious | Not serious | Not serious | None | ⨁◯◯◯ |
| **Food groups** | | | | | | | | | | |
| Dairy | Matia-martin et al. (2019) | Per 'increment' increase | 5 | Prospective cohort | Serious^ag^ | Very serious^d^ | Not serious | Not serious | None | ⨁◯◯◯ |
| Milk | Hidayat et al. (2020) | Increase of 1 glass/day | 7 | Prospective cohort | Serious^g^ | Serious^b^ | Not serious | Not serious | None | ⨁◯◯◯ |
| Yogurt | Hidayat et al. (2020) | High vs low | 4 | Prospective cohort | Serious^g^ | Not serious | Not serious | Not serious | None | ⨁◯◯◯ |
| Cheese | Hidayat et al. (2020) | High vs low | 4 | Prospective cohort | Serious^g^ | Very serious^d^ | Not serious | Serious^e^ | None | ⨁◯◯◯ |
| Fruits | Luo et al. (2016) | High vs low | 5 | Prospective cohort | Serious^c^ | Serious^b^ | Not serious | Serious^e^ | None | ⨁◯◯◯ |
| Vegetables | Luo et al. (2016) | High vs low | 5 | Prospective cohort | Serious^c^ | Serious^b^ | Not serious | Not serious | None | ⨁◯◯◯ |
| Fruits and vegetables | Brondani et al. (2019) | High vs low | 5 | Prospective cohort | Not serious | Not serious* | Not serious | Not serious | None | ⨁⨁◯◯ |
| Tea | Sheng et al. (2013) | High vs low | 3 | Prospective cohort | Very serious^f^ | Not serious | Not serious | Serious^e^ | None | ⨁◯◯◯ |
| Coffee | Li and Xu (2013) | Per cup increase/day | 4 | Prospective cohort | Very serious^f^ | Serious^b^ | Not serious | Not serious | None | ⨁◯◯◯ |
| Alcohol | Zhang et al. (2014) | Any vs none | 18 | Prospective cohort | Very serious^f^ | Very serious^d^ | Not serious | Not serious | None | ⨁◯◯◯ |
|  |  | Light vs none | 7 | Prospective cohort | Very serious^f^ | Not serious | Not serious | Not serious | None | ⨁◯◯◯ |
|  |  | Moderate vs none | 7 | Prospective cohort | Very serious^f^ | Serious^b^ | Not serious | Not serious | None | ⨁◯◯◯ |
|  |  | Heavy vs none | 3 | Prospective cohort | Very serious^f^ | Not serious | Not serious | Not serious | None | ⨁◯◯◯ |
| Wine | Zhang et al. (2014) | Any vs no alcohol | 4 | Prospective cohort | Very serious^f^ | Not serious | Not serious | Not serious | None | ⨁◯◯◯ |
| Beer | Zhang et al. (2014) | Any vs no alcohol | 4 | Prospective cohort | Very serious^f^ | Serious^b^ | Not serious | Serious^e^ | None | ⨁◯◯◯ |
| Liquor | Zhang et al. (2014) | Any vs no alcohol | 4 | Prospective cohort | Very serious^f^ | Not serious | Not serious | Serious^e^ | None | ⨁◯◯◯ |
| **Macronutrients** | | | | | | | | | | |
| Dietary protein | Wu et al. (2015) | High vs low | 6 | Prospective cohort | Serious^a^ | Not serious | Not serious | Not serious | None | ⨁◯◯◯ |
| Animal protien | Wu et al. (2015) | High vs low | 4 | Prospective cohort | Serious^a^ | Not serious* | Not serious | Serious^e^ | None | ⨁◯◯◯ |
| Vegetable protein | Wu et al. (2015) | High vs low | 3 | Prospective cohort | Serious^a^ | Not serious* | Not serious | Serious^e^ | None | ⨁◯◯◯ |
| **Micronutrients** | | | | | | | | | | |
| Dietary vitamin C | Malmir et al. (2018b) | High vs low | 3 | Prospective cohort | Serious^c^ | Not serious | Not serious | Serious^e^ | None | ⨁◯◯◯ |
| Dietary vitamin A | Zhang et al. (2017) | High vs low | 3 | Prospective cohort | Serious^c^ | Not serious | Not serious | Not serious | None | ⨁◯◯◯ |
| Dietary carotenoids | Xu et al. (2017) | High vs low | 2 | Prospective cohort | Serious^c^ | Not serious | Not serious | Serious^e^ | None | ⨁◯◯◯ |
| Dietary ALA | Sadeghi et al. (2019) | High vs low | 3 | Prospective cohort | Serious^c^ | Serious^b^ | Not serious | Not serious | None | ⨁◯◯◯ |
| Dietary EPA + DHA | Sadeghi et al. (2019) | High vs low | 4 | Prospective cohort | Serious^c^ | Not serious | Not serious | Not serious | None | ⨁◯◯◯ |
| Dietary calcium | Bischoff-Ferrari et al. (2007) | Increase of 300 mg/d | 4 | Prospective cohort | Very serious^f^ | Not serious | Not serious | Not serious | None | ⨁◯◯◯ |
| Dietary retinol | Zhang et al. (2017) | High vs low | 4 | Prospective cohort | Serious^c^ | Serious^b^ | Not serious | Not serious | None | ⨁◯◯◯ |
| Dietary a-carotene | Xu et al. (2017) | High vs low | 2 | Prospective cohort | Serious^c^ | Serious^b^ | Not serious | Serious^e^ | None | ⨁◯◯◯ |
| Dietary b-carotene | Zhang et al. (2017) | High vs low | 2 | Prospective cohort | Serious^c^ | Serious^b^ | Not serious | Serious^e^ | None | ⨁◯◯◯ |
| Dietary b-cryptoxanthin | Xu et al. (2017) | High vs low | 2 | Prospective cohort | Serious^c^ | Not serious | Not serious | Serious^e^ | None | ⨁◯◯◯ |
| Dietary lycopene | Xu et al. (2017) | High vs low | 2 | Prospective cohort | Serious^c^ | Not serious | Not serious | Serious^e^ | None | ⨁◯◯◯ |
| Dietary lutein/zeaxanthin | Xu et al. (2017) | High vs low | 2 | Prospective cohort | Serious^c^ | Not serious | Not serious | Not serious | None | ⨁◯◯◯ |
| Antioxidant vitamins | Zhou et al. (2020) | High vs low | 9 | Prospective cohort | Serious^c^ | Serious^b^ | Not serious | Serious^e^ | None | ⨁◯◯◯ |

a: rated down by one level due to possible bias from the method of ascertainment of exposure and outcome data in primary studies.

b: rated down by one level due to high, significant unexplained heterogeneity (I^2^>50%, p < 0.05) or different direction of effects in different studies, with minimal or no overlap of confidence intervals.

c: rated down by one level because the risk of bias for each domain was not reported, thus remains unclear.

d: rated down by two levels due to high significant heterogeneity and minimal overlap of confidence intervals between studies, with different directions of effect.

e: rated down by one level because the confidence intervals of the meta-analytic effect overlapped 1 and failed to exclude the possibility of an appreciable benefit or harm (relative risk reduction or increase of 25% was used as a threshold as recommended in the GRADE handbook).

f: rated down by two levels because no risk of bias assessment was reported.

g: rated down by one level due to possible bias from inadequate follow-up.

*: near-significant p value for heterogeneity (p < 0.1).
