# Supplementary material for: Dietary risk factors for hip fracture in adults: An umbrella review of meta-analyses of prospective cohort studies
Source: PLoS One. 2021 Nov 10;16(11):e0259144. doi: 10.1371/journal.pone.0259144 (PMC8580223; doi:10.1371/journal.pone.0259144)
Supplement: S8 Table — (DOCX) [file pone.0259144.s008.docx]

**S8 Table: PRISMA 2020 checklist for abstracts.**

| **Section/Topic** | **Item no.** | **Checklist item** | **Line number reported** |
| --- | --- | --- | --- |
| **TITLE** | | | |
| Title | 1 | Identify the report as a systematic review. | 1-2 |
| **BACKGROUND** | | | |
| Objectives | 2 | Provide an explicit statement of the main objective(s) or question(s) the review addresses. | 12 13 |
| **METHODS** | | | |
| Eligibility criteria | 3 | Specify the inclusion and exclusion criteria for the review. | 15-17 |
| Information sources | 4 | Specify the information sources (e.g. databases, registers) used to identify studies and the date when each was last searched. | 18-19 |
| Risk of bias | 5 | Specify the methods used to assess risk of bias in the included studies. | 20-22 |
| Synthesis of results | 6 | Specify the methods used to present and synthesize results | 20-22 |
| **RESULTS** | | | |
| Included studies | 7 | Give the total number of included studies and participants and summarise relevant characteristics of studies. | 23-30 |
| Synthesis of results | 8 | Present results for main outcomes, preferably indicating the number of included studies and participants for each. If meta-analysis was done, report the summary estimate and confidence/credible interval. If comparing groups, indicate the direction of the effect (i.e. which group is favoured). | 23-30 |
| **DISCUSSION** | | | |
| Limitations of evidence | 9 | Provide a brief summary of the limitations of the evidence included in the review (e.g. study risk of bias, inconsistency and imprecision). | 31-35 |
| Interpretation | 10 | Provide a general interpretation of the results and important implications. | 31-35 |
| **OTHER** | | | |
| Funding | 11 | Specify the primary source of funding for the review | N/A |
| Registration | 12 | Provide the register name and registration number. | 36 |
