# Supplementary material for: Dietary risk factors for hip fracture in adults: An umbrella review of meta-analyses of prospective cohort studies
Source: PLoS One. 2021 Nov 10;16(11):e0259144. doi: 10.1371/journal.pone.0259144 (PMC8580223; doi:10.1371/journal.pone.0259144)
Supplement: Attachment — Submitted filename: UR review Report .docx [file pone.0259144.s009.docx]

REPORT

Title: `**Dietary risk factors for hip fracture: an umbrella review of Meta-analyses of prospective cohort studies**`

- **Title:**
- In articulating a title of this particular Umbrella Review, the population of interest is not evident.
- Is it a review of Meta-analysis (which is only 2 out of 34-exposures)?
- Suggestion: Dietary risk factors for hip fracture *in adults*.

: An Umbrella Review of Prospective cohort studies.

- **Objective:**
- `To summarize the totality of evidence regarding dietary factors and hip fracture i*ncidence* in adults, and to evaluate the methodological quality of systematic reviews and meta-analyses
- *Is it appropriate to use the Term `Incidence`?*
- *`Methodological quality of systematic reviews`* -This is the analysis procedure to be performed during the process and not the objective to be set
- Suggestion: To summarize the *`totality of evidence* regarding dietary factors for hip fracture` in adults
- **Methodology and synthesis:**

``The quality of evidence for all associations except combined fruits and vegetables intake and hip fracture incidence was downgraded from low to very low due to a serious or very serious risk of bias *amongst primary studies``*

- The purpose of UR quality assessment is to assess methodological quality, risk of bias, and reporting quality of reviews, and does not involve retrieving the quality of primary studies??
- Any modifications to the JBI umbrella review method?
- **Result;**
- `The quality of evidence (GRADE) was low for an inverse association between hip fracture incidence and intake of fruits and vegetables combined (adjusted relative risk for higher vs lower intakes: 0.92 [95% confidence interval: 0.87 to 0.98]), and very low for the remaining thirty-three exposures. Adjusted summary effect estimates and the quality of evidence for each exposure regarding risk of hip fracture are summarized.

**Question:**

- Wasn`t it necessary to show cumulative risk factor (effect size)? By forest plot, Summary ROC (SROC) curves…or other ways of addressing heterogeneity of the Meta-analysis. Because, if the differences are not the result of chance, then we need to be cautious in interpreting the results of the meta-analysis.
- Nineteen meta-analyses had an I^2^ value above 50%, and heterogeneity was significant for the 14 exposures. If studies are heterogeneous, is it certain or appropriate to synthesize the respective studies into meta-analysis??
- Does this UR not require approval of IRBs?
